# Lung Cancer Risk Prediction Nomogram in Nonsmoking Chinese Women: Retrospective Cross-sectional Cohort Study

**DOI:** 10.2196/41640

**Published:** 2023-01-06

**Authors:** Lanwei Guo, Qingcheng Meng, Liyang Zheng, Qiong Chen, Yin Liu, Huifang Xu, Ruihua Kang, Luyao Zhang, Shuzheng Liu, Xibin Sun, Shaokai Zhang

**Affiliations:** 1 Department of Cancer Epidemiology and Prevention, Henan Engineering Research Center of Cancer Prevention and Control, Henan International Joint Laboratory of Cancer Prevention The Affiliated Cancer Hospital of Zhengzhou University & Henan Cancer Hospital Zhengzhou China; 2 Department of Radiology The Affiliated Cancer Hospital of Zhengzhou University & Henan Cancer Hospital Zhengzhou China

**Keywords:** lung cancer, risk model, forecasting, validation, female, nonsmokers

## Abstract

**Background:**

It is believed that smoking is not the cause of approximately 53% of lung cancers diagnosed in women globally.

**Objective:**

The study aimed to develop and validate a simple and noninvasive model that could assess and stratify lung cancer risk in nonsmoking Chinese women.

**Methods:**

Based on the population-based Cancer Screening Program in Urban China, this retrospective, cross-sectional cohort study was carried out with a vast population base and an immense number of participants. The training set and the validation set were both constructed using a random distribution of the data. Following the identification of associated risk factors by multivariable Cox regression analysis, a predictive nomogram was developed. Discrimination (area under the curve) and calibration were further performed to assess the validation of risk prediction nomogram in the training set, which was then validated in the validation set.

**Results:**

In sum, 151,834 individuals signed up to take part in the survey. Both the training set (n=75,917) and the validation set (n=75,917) were comprised of randomly selected participants. Potential predictors for lung cancer included age, history of chronic respiratory disease, first-degree family history of lung cancer, menopause, and history of benign breast disease. We displayed 1-year, 3-year, and 5-year lung cancer risk–predicting nomograms using these 5 factors. In the training set, the 1-year, 3-year, and 5-year lung cancer risk areas under the curve were 0.762, 0.718, and 0.703, respectively. In the validation set, the model showed a moderate predictive discrimination.

**Conclusions:**

We designed and validated a simple and noninvasive lung cancer risk model for nonsmoking women. This model can be applied to identify and triage people at high risk for developing lung cancers among nonsmoking women.

## Introduction

China has the most lung cancer death cases around the world in 2020. In 2020, according to estimates provided by the International Agency for Research on Cancer, there were approximately 1.80 million cases of deadly lung cancer globally. China accounted for 39.8% of these cases [1]. In China, the continuous rise in lung cancer deaths during the past 2 decades was attributed to the rising prevalence of lung cancer in women [2]. Additionally, 50% or more of lung cancers in women in Southeast Asia were diagnosed in nonsmokers [3-5]. Most of the Chinese lung cancer cases were reported to be clinically progressed in 2012-2014, with 64.6% of them being stage III-IV lung cancers [6]. The lung cancer survival rate in China, which was defined as 5 years as standard, grew up to 20% between 2003 and 2015 [7]. The prognosis of lung cancer is strongly associated with the stage in which it was detected; the 5-year survival rate ranges from 0% in cases detected in patients with stage IV cancer to >80% in cases detected in stage I and whose patients underwent surgery [8].

Started in 2002, the National Lung Screening Trial indicated that low-dose computed tomography screening may decrease lung cancer deaths by 20% [9]. However, this project only screened people (41% women) at high risk for lung cancer based on age and smoking history (aged 55-74 years, smoked no fewer than 30 pack-years, and had no more than 15 years of having quit smoking). Women in China have their own characteristics of lung cancer risk factor exposure and incidence patterns, the most critical of which is that although the smoking rate among women is much lower than that of high-income countries such as the United States (2.4% in China and 23.6% in the United States), the lung cancer frequencies are relatively similar (22.8/100,000 in China and 30.8/100,000 in the United States, based on the standardized lung cancer incidence rate of the world population) [10,11]. This finding shows that the existing worldwide guidelines for lung cancer screening focused on smoking as the primary predictor for high-risk individuals, which would be inappropriate for Chinese women, particularly for nonsmoking women. Therefore, determining a way that accurately forecasts the risk of lung cancer in nonsmoking women and directing them toward the more cost-effective low-dose computed tomography screening is a feasible method for achieving efficient early diagnosis and treatment of lung cancer.

Earlier research has developed numerous lung cancer risk predictive models related to specific population demographics [12-41]; however, few of the predictive methods focused on nonsmoking women in mainland China [42]. Consequently, the development of lung cancer risk predicting tools for nonsmoking Chinese women according to consistently established risk factors in earlier studies has become a top goal [43]. Nevertheless, this goal is demanding and difficult. In contrast to the findings of lung cancer caused by tobacco, there are no identified risk variables for the progression of lung cancer in nonsmoking women. Although other risk factors were suggested, their relative importance varies greatly between geographical locations [3,4,44,45]. It was observed that the Prostate, Lung, Colorectal, and Ovarian Cancer Screening Trial (PLCO) models, which included nearly 2000 Asian nonsmokers and only 7 cases of lung cancer, could be inapplicable to Asian nonsmokers [46]. Among the nonsmokers who participated in the PLCO study (n=65,711), none of them had a 6-year risk that was greater than 0.0151.

On the basis of the Cancer Screening Program in Urban China (CanSPUC), we created such a model [47]. In this paper, we aimed to create and internally validate a lung cancer risk predicting model for nonsmoking Chinese women, with the focus on established risk factors for lung cancer routinely available in general cancer-screening settings.

## Methods

### Data Source and Subjects

This retrospective, cross-sectional cohort study was carried out inside the scope of CanSPUC, a continuing statewide cancer-screening program for China’s urban population. CanSPUC is designed to detect the 5 most common malignancies, including lung cancer, colorectal cancer, upper gastrointestinal cancer, liver cancer, and female breast cancer. The CanSPUC approach was detailed in previous studies [47,48]. All of the qualified subjects were questioned by highly skilled staff to gather information about their exposure to risk variables and to assess their cancer risk using a specific cancer risk score system. The household registration system was used in local communities to identify eligible permanent residents who were aged 40-74 years and asymptomatic for lung cancer with no history of cancer diagnosis. Individuals who were unable to give informed consent, had a medical disability and were unlikely to complete curative lung cancer surgery, had a history of lung cancer, had received treatment for or had evidence of any cancer within the past 5 years (with the exception of nonmelanoma skin cancer and most in situ carcinomas), or had symptoms suggestive of lung cancer (including unexplained weight loss of >7.5 kg within the past 12 months or unexplained hemoptysis) were not eligible to participate. In October 2013, CanSPUC was implemented in Henan province, which encompassed 8 cities with complete cancer registration data (Zhengzhou, Zhumadian, Anyang, Luoyang, Nanyang, Jiaozuo, Puyang, and Xinxiang). We examined the data collected over the first 6 years (from October 2013 to October 2019) in the Henan province for our research. Only nonsmoking women were included in this investigation.

### Ethics Approval

The Ethics Committee of the Affiliated Cancer Hospital of Zhengzhou University and Henan Cancer Hospital evaluated and authorized the research (no.2021-KY-0028-001). Our sample was drawn from retrospective encounters documented in the electronic health record; these data were deidentified for both sets of analyses and did not require informed consent.

### Outcome, Variables, and Measurements

All new cases of lung cancer were identified by matching with the cancer registry database in Henan province, China (by unique ID number), and histologically confirmed between October 1, 2013, and March 10, 2020. In Henan province, records of lung cancer are first submitted to local cancer registries by the hospitals and medical institutions and then submitted to the Henan Provincial Central Cancer Registry of China by the local cancer registries. The International Classification of Diseases, Tenth Revision was used to classify newly diagnosed lung malignancies by site. Lung cancers were identified by the International Classification of Diseases, Tenth Revision code of C33-C34. To find possible lung cancer risk variables, self-reported information was collected (Textbox 1).

**Self-reported information collected.**

1. **Demographic factors, such as age, ethnicity, educational status, marital status, height, and body weight**
  - A low-educational level was defined as elementary school or less, a medium educational level as junior or senior high school, and a high-educational level as college or above
  - According to the “Guidelines for the Prevention and Control of Overweight and Obesity in Chinese Adults,” BMI was dependent on the individual’s height as well as weight and segmented to “<18.5 kg/m^2^,” “18.5-23.9 kg/m^2^,” “24.0-27.9 kg/m^2^,” and “≥28.0 kg/m^2^” categories [49]
2. **Dietary habit**
  - Dietary intake of the following food in the past 2 years: vegetables (green-leafy plants and fungi except potatoes, sweet potatoes, and starches) <2.5 kg/week or ≥2.5 kg/week; roughage (all other grains except for white flour and rice) <0.5 kg/week or ≥0.5 kg/week; and fruit <1.25 kg/week or ≥1.25 kg/week. The weight of the food was measured prior to cooking
3. **Living environment, behavior, and habits**
  - Cooking oil fume exposure: exposure is considered as “none or a little” if chimneys, fume extractors, or smokeless pots were used during cooking; otherwise, it was considered as “a lot”
  - Passive smoking: regular living or employment in an enclosed area where people routinely smoke was regarded as “yes”; otherwise, it was regarded as “no”
  - Alcohol consumption: “current” referred to those who had consumed alcohol at least once weekly on average for more than 6 months; “former” referred to those who had ceased drinking; “never” referred to those who had never consumed alcohol
  - Physical activity: swimming; taijiquan, qigong, or walking; long range running; aerobics; sporting events (such as basketball, table tennis, badminton, etc); Yangko dancing or fast walking; and other physical activities (such as mountains climbing, rope jumping, and shuttlecock kicking). Subjects who engage in at least three sessions of practice weekly for a total of ≥90 minutes weekly were classified as engaging in “heavy physical activity”; otherwise, they were classified as engaging in “moderate or no physical activity”
4. **Psychology and emotions, such as a history of serious trauma and more than 6 months of mental depression**
  - Serious trauma was described as a major illness or death of a family member, family conflict and separation, significant loss of property, unexpected job loss, severe unexpected physical injury, violent danger, etc
5. **Comorbidities, such as chronic respiratory disease, tuberculosis, chronic bronchitis, emphysema, asthma bronchiectasis, hypertension, hyperlipidemia, and diabetes**
  - Every self-reported case of comorbidity required an evaluation from a professional medical facility
6. **Family history of lung cancer**
  - Whether first-degree relatives, second-degree relatives, or third-degree relatives had lung cancer or not
7. **Physiology and fertility**
  - Including age of menarche (<12 years or ≥12 years), menopause (yes or no), fertility status (yes or no), lactation status (yes or no), history of benign breast illness (yes or no), and a history of reproductive system surgery (yes or no)

### Statistical Analysis

To contrast the profiles of those who have lung cancer and those without cancer, descriptive statistics, presented as percentages for categorical data, were used. Chi-square tests were used to examine the univariate correlation between baseline characteristics and lung cancer progression. For continuous variables, mean (SD) or median (IQR) were used.

In this investigation, the integrated model was applied to generate a nomogram to measure the 1-year, 3-year, and 5-year estimations of the lung cancer risk in the training set, according to the independently prognostic variables using the stepwise multivariable Cox regression (*P*_entry_=.15 and *P*_stay_=.10). The calibration curve was used to determine the nomogram’s validity. By applying 50% and 84% quantiles, the risk predictions were grouped into the low-risk group, medium-risk group, and high-risk group, as suggested previously [50]. As per the risk prediction model, Kaplan-Meier curves were displayed for the low-risk group, medium-risk group, and high-risk group for lung cancer. The log-rank analyses were performed to compare the 3 curves. Receiver operating characteristic curves and the area under the curve (AUC) were used to quantify the prediction performance of 1-year, 3-year, and 5-year lung cancer risk estimations in the training set and validation set. By comparing observed and predicted probabilities, the bootstrap sampling method was used to evaluate the calibration of the current model.

All statistical analysis was carried out via R (version 4.0.3; R Foundation for Statistical Computing) and SAS (version 9.4; SAS Institute) software. The nomogram was drawn using the *rms* package. The receiver operating characteristic curves were drawn by using the *survivalROC* package. Using the *ggplot2* package, a calibration curve was created. All of the tests were done using 2-tailed hypotheses, and *P*<.05 was determined to be statistically significant.

## Results

### Characteristics of the Study Population

**Figure 1:**
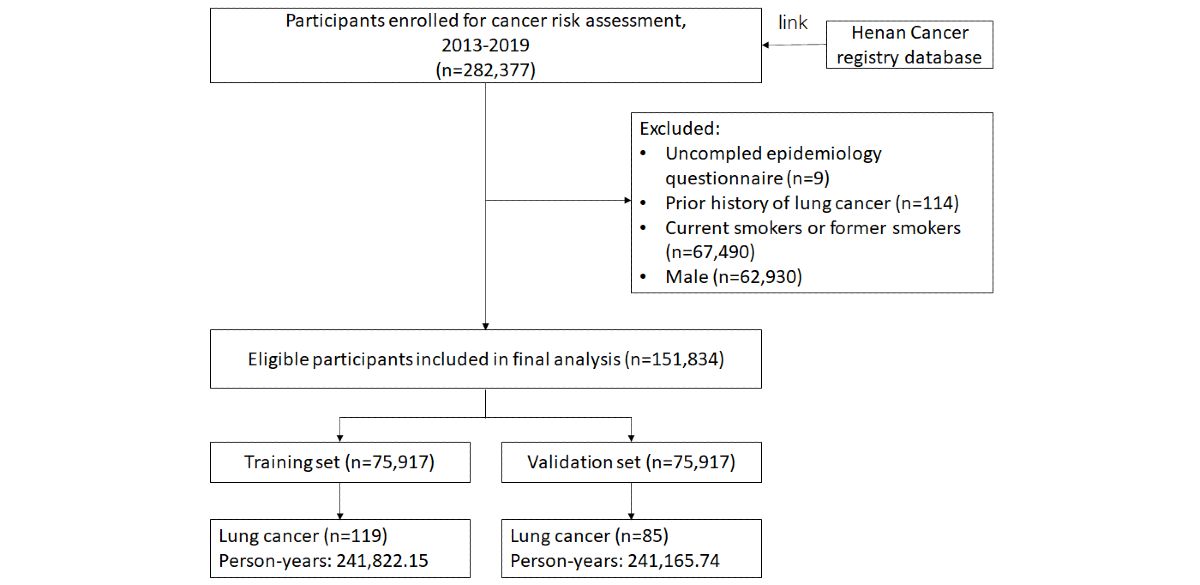
Flow chart of participants included in this analysis.

This research consisted of a total of 151,834 qualified participants with an average age of 55.34 (SD 8.65) years. The subjects were randomly separated into a training set of 75,917 and a validation set of 75,917 (Figure 1). By March 2020, 204 lung cancer cases occurred within 151,834 subjects, resulting in an incident density of 42.24 per 100,000 person-years. Lung cancer cases were more frequent in those who were older (*P*<.001), had a history of respiratory illness (*P*=.001), had a first-degree family history of lung cancer (*P*=.02), and had menopause (*P*<.001). Extra features are shown in Table 1 and Table S1 in Multimedia Appendix 1.

**Table 1:** Comparison of baseline characteristics between the non–lung cancer and lung cancer groups using chi-square test in the training set.

Variables				Total (N=75,917)^a^		Non–lung cancer^b^	Lung cancer^b^		*χ*^2^ (*df*)			*P* value	
All participants				75,917 (100)		75,798 (99.84)	119 (0.16)						
Person-years, median (IQR)				2.95 (1.73-4.83)		2.95 (1.73-4.83)	1.56 (0.83-2.38)						
**Demographic characteristics**													
	Age (years), mean (SD)			55.37 (8.65)		55.36 (8.65)	60.37 (7.20)						
	**Age (years), n (%)**									47.96 (6)			*<.001* ^c^
		40-44	9226 (12.15)		9221 (99.95)		5 (0.05)						
		45-49	13,558 (17.86)		13,551 (99.95)		7 (0.05)						
		50-54	14,389 (18.95)		14,376 (99.91)		13 (0.09)						
		55-59	11,857 (15.62)		11,838 (99.84)		19 (0.16)						
		60-64	12,927 (17.03)		12,889 (99.71)		38 (0.29)						
		65-69	10,181 (13.41)		10,151 (99.71)		30 (0.29)						
		70-74	3779 (4.98)		3772 (99.81)		7 (0.19)						
	**Race, n (%)**									0.05 (1)			.83
		Han nationality	74,431 (98.04)		74,314 (99.84)		117 (0.16)						
		Others	1486 (1.96)		1484 (99.87)		2 (0.13)						
	**Education^d^, n (%) **									0.12 (2)			.94
		Low	16,139 (21.26)		16,115 (99.85)		24 (0.15)						
		Medium	49,922 (65.76)		49,842 (99.84)		80 (0.16)						
		High	9856 (12.98)		9841 (99.85)		15 (0.15)						
	**Marriage, n (%)**									1.89 (1)			.17
		Unmarried, divorce, or widowed	3193 (4.21)		3191 (99.94)		2 (0.06)						
		Married	72,724 (95.79)		72,607 (99.84)		117 (0.16)						
	**BMI (kg/m^2^), n (%)**									1.84 (3)			.61
		<18.5	1133 (1.49)		1133 (100)		0 (0)						
		18.5-24.0	35,445 (46.69)		35,388 (99.84)		57 (0.16)						
		24.0-28.0	30,729 (40.48)		30,681 (99.84)		48 (0.16)						
		≥28.0	8610 (11.34)		8596 (99.84)		14 (0.16)						
**Dietary habit, n (%)**													
	**Vegetables intake (kg/week)**									0.01 (1)			.92
		≥2.5	39,282 (51.74)		39,221 (99.84)		61 (0.16)						
		<2.5	36,635 (48.26)		36,577 (99.84)		58 (0.16)						
	**Fruit intake (kg/week)**									0.71 (1)			.40
		≥1.25	43,683 (57.54)		43,610 (99.83)		73 (0.17)						
		<1.25	32,234 (42.46)		32,188 (99.86)		46 (0.14)						
	**Roughage intake (kg/week)**									0.64 (1)			.42
		≥0.5	51,713 (68.12)		51,636 (99.85)		77 (0.15)						
		<0.5	24,204 (31.88)		24,162 (99.83)		42 (0.17)						
**Living environment, behavior, and habits, n (%)**													
	**Cooking oil fume exposure**									0.05 (1)			.82
		None or a little	65,819 (86.70)		65,715 (99.84)		104 (0.16)						
		A lot	10,098 (13.3)		10,083 (99.85)		15 (0.15)						
	**Passive smoking**									0.63 (1)			.43
		No	49,045 (64.6)		48,964 (99.83)		81 (0.17)						
		Yes	26,872 (35.4)		26,834 (99.86)		38 (0.14)						
	**Alcohol drinking**									0.11 (2)			.95
		Never	71,567 (94.27)		71,454 (99.84)		113 (0.16)						
		Current	3647 (4.8)		3642 (99.86)		5 (0.14)						
		Former	703 (0.93)		702 (99.86)		1 (0.14)						
	**Physical activity**									3.19 (1)			.07
		Moderate or no	40,014 (52.71)		39,961 (99.87)		53 (0.13)						
		Heavy	35,903 (47.29)		35,837 (99.82)		66 (0.18)						
**Psychology and emotions, n (%)**													
	**History of a severe trauma**									1.22 (1)			.27
		No	65,199 (85.88)		65,101 (99.85)		98 (0.15)						
		Yes	10,718 (14.12)		10,697 (99.8)		21 (0.2)						
	**Mental depression for over 6 months**									0.00 (1)			.98
		No	64,379 (84.8)		64,278 (99.84)		101 (0.16)						
		Yes	11,538 (15.2)		11,520 (99.84)		18 (0.16)						
**Comorbidities, n (%)**													
	**History of chronic respiratory disease**									11.53 (1)			*.001*
		No	64,070 (84.39)		63,983 (99.86)		87 (0.14)						
		Yes	11,847 (15.61)		11,815 (99.73)		32 (0.27)						
	**History of tuberculosis**									1.24 (1)			.27
		No	74,895 (98.65)		74,779 (99.85)		116 (0.15)						
		Yes	1022 (1.35)		1019 (99.71)		3 (0.29)						
	**History of chronic bronchitis**									3.44 (*1*)			.06
		No	66,728 (87.9)		66,630 (99.85)		98 (0.15)						
		Yes	9189 (12.1)		9168 (99.77)		21 (0.23)						
	**History of emphysema**									3.21 (1)			.07
		No	75,204 (99.06)		75,088 (99.85)		116 (0.15)						
		Yes	713 (0.94)		710 (99.58)		3 (0.42)						
	**History of asthma bronchiectasis**									1.27 (1)			.26
		No	73,473 (96.78)		73,360 (99.85)		113 (0.15)						
		Yes	2444 (3.22)		2438 (99.75)		6 (0.25)						
	**History of hypertension**									1.66 (1)			.20
		No	60,976 (80.32)		60,886 (99.85)		90 (0.15)						
		Yes	14,941 (19.68)		14,912 (99.81)		29 (0.19)						
	**History of hyperlipidemia**									1.67 (1)			.20
		No	63,309 (83.39)		63,215 (99.85)		94 (0.15)						
		Yes	12,608 (16.61)		12,583 (99.8)		25 (0.2)						
	**History of diabetes**									0.00 (1)			.98
		No	70,767 (93.22)		70,656 (99.84)		111 (0.16)						
		Yes	5150 (6.78)		5142 (99.84)		8 (0.16)						
**First-degree family history of lung cancer, n (%)**										5.15 (1)			*.02*
	No			69,955 (92.15)		69,852 (99.85)	103 (0.15)						
	Yes			5962 (7.85)		5946 (99.73)	16 (0.27)						
**Physiology and fertility, n (%)**													
	**Age of menarche (years)**									0.34 (1)			.56
		<12	1910 (2.52)		1908 (99.9)		2 (0.1)						
		≥12	74,007 (97.48)		73,890 (99.84)		117 (0.16)						
	**Menopause**									29.26 (1)			*<.001*
		No	26,927 (35.47)		26,913 (99.95)		14 (0.05)						
		Yes	48,990 (64.53)		48,885 (99.79)		105 (0.21)						
	**Fertility status**									1.67 (1)			.20
		No	1047 (1.38)		1047 (100)		0 (0)						
		Yes	74,870 (98.62)		74,751 (99.84)		119 (0.16)						
	**Lactation status**									0.06 (1)			.80
		No	4233 (5.58)		4227 (99.86)		6 (0.14)						
		Yes	71,684 (94.42)		71,571 (99.84)		113 (0.16)						
	**History of benign breast disease**									3.61 (1)			.06
		No	53,977 (71.1)		53,883 (99.83)		94 (0.17)						
		Yes	21,940 (28.9)		21,915 (99.89)		25 (0.11)						
	**History of reproductive system surgery**									1.75 (1)			.19
		No	60,480 (79.67)		60,391 (99.85)		89 (0.15)						
		Yes	15,437 (20.33)		15,407 (99.81)		30 (0.19)						

^a^Percentages in this column have denominators of N=75,917.

^b^Percentages in these columns have the n value in the “Total” column in the same row as the denominators.

^c^Italicized values indicate statistical singificance.

^2^Low=primary school or below; medium=junior or senior high school; high=undergraduate degree or above.

### Development of the Lung Cancer Risk Assessment Model

**Figure 2:**
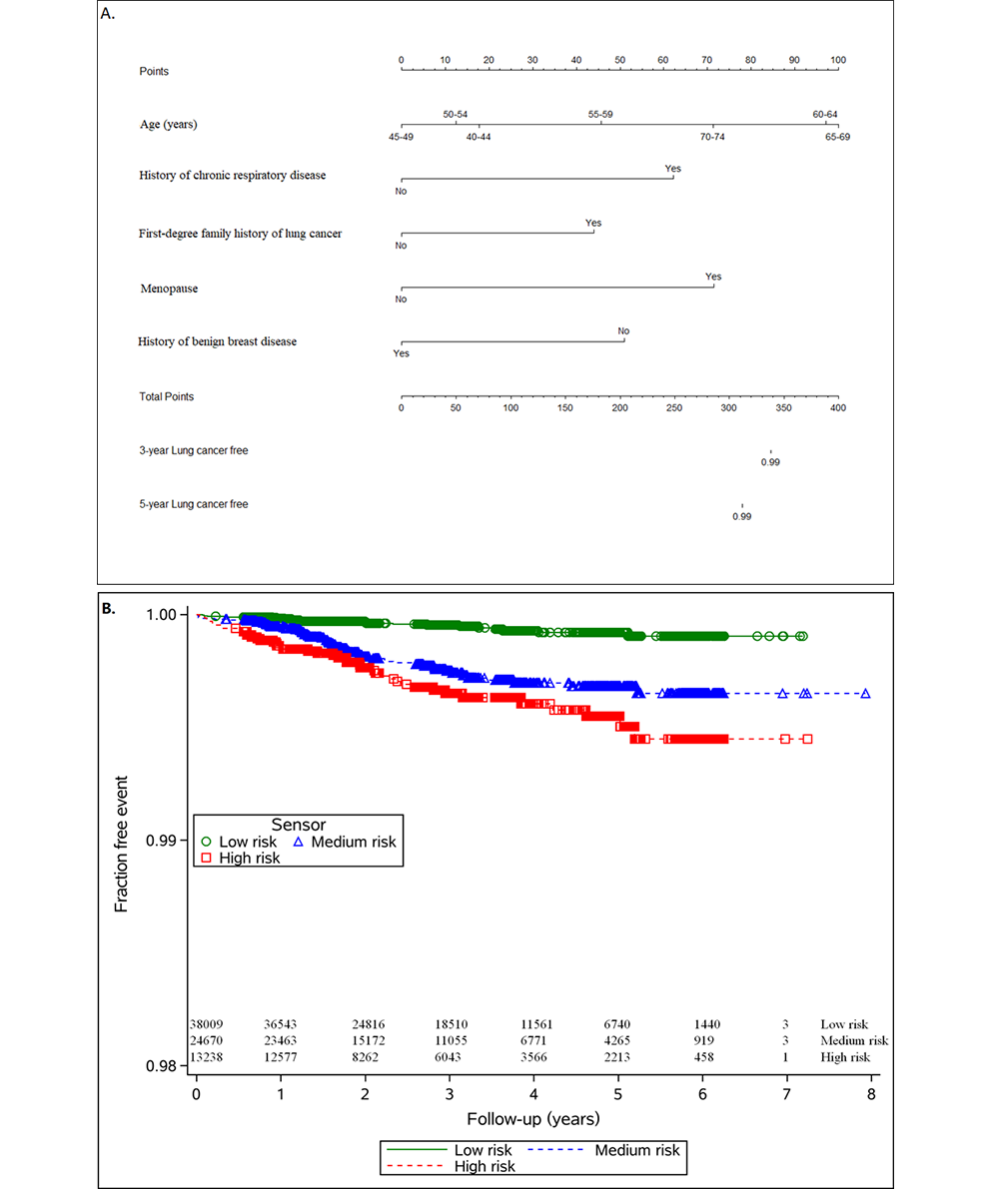
(A) Nomogram to calculate the personal 1-year, 3-year, and 5-year risk of lung cancer, and (B) the lung cancer incidence across different cancer risk categories.

Table 2 displays the hazard ratios (HRs) with its 95% CI for every indicator. In the training set, age (≥55 years: HR 1.34, 95% CI 0.38-4.80; ≥60 years: HR 2.33, 95% CI 0.67-8.11; ≥65 years: HR 2.41, 95% CI 0.69-8.49; ≥70 years: HR 1.79, 95% CI 0.43-7.40), history of chronic respiratory disease (HR 1.94, 95% CI 1.24-3.04), first-degree family history of lung cancer (HR 1.60, 95% CI 0.91-2.83), menopause (HR 2.16, 95% CI 0.90-5.19), and history of benign breast disease (HR 0.58, 95% CI 0.36-0.94) were independent risk factors for lung cancer. Consequently, we applied these parameters to construct the model. We drew 1-year, 3-year, and 5-year risk–predicting nomograms for lung cancer (Figure 2A).

**Table 2:** Multivariable Cox regression prediction model of lung cancer risk in the training set.

Variables		β coefficient	SE	HR^a^ (95% CI)	*χ*^2^ (*df*)	*P* value
**Age (years)**						
	40-44	N/A^b^	N/A	1.00	N/A	N/A
	45-49	–0.19	0.59	0.83 (0.26-2.64)	0.10 (1)	.75
	50-54	–0.06	0.62	0.94 (0.28-3.19)	0.01 (1)	.93
	55-59	0.30	0.65	1.34 (0.38-4.80)	0.21 (1)	.65
	60-64	0.85	0.64	2.33 (0.67-8.11)	1.78 (1)	.18
	65-69	0.88	0.64	2.41 (0.69-8.49)	1.89 (1)	.17
	70-74	0.58	0.72	1.79 (0.43-7.40)	0.65 (1)	.42
**History of chronic respiratory disease**						
	No	N/A	N/A	1.00	N/A	N/A
	Yes	0.66	0.23	1.94 (1.24-3.04)	8.45 (1)	*.004* ^c^
**First-degree family history of lung cancer**						
	No	N/A	N/A	1.00	N/A	N/A
	Yes	0.47	0.29	1.60 (0.91-2.83)	2.63 (1)	.11
**Menopause**						
	No	N/A	N/A	1.00	N/A	N/A
	Yes	0.77	0.45	2.16 (0.90-5.19)	2.95 (1)	.09
**History of benign breast disease**						
	No	N/A	N/A	1.00	N/A	N/A
	Yes	–0.55	0.25	0.58 (0.36-0.94)	4.97 (1)	*.03*

^a^HR: hazard ratio.

^b^N/A: not applicable.

^c^Italicized values indicate statistical significance.

### Predictive Performance of the Model

The risk predictions were categorized into low-risk, medium-risk, and high-risk categories, and a log-rank test revealed significant differences across the 3 groups (Figure 2B; *P*<.001).

**Figure 3:**
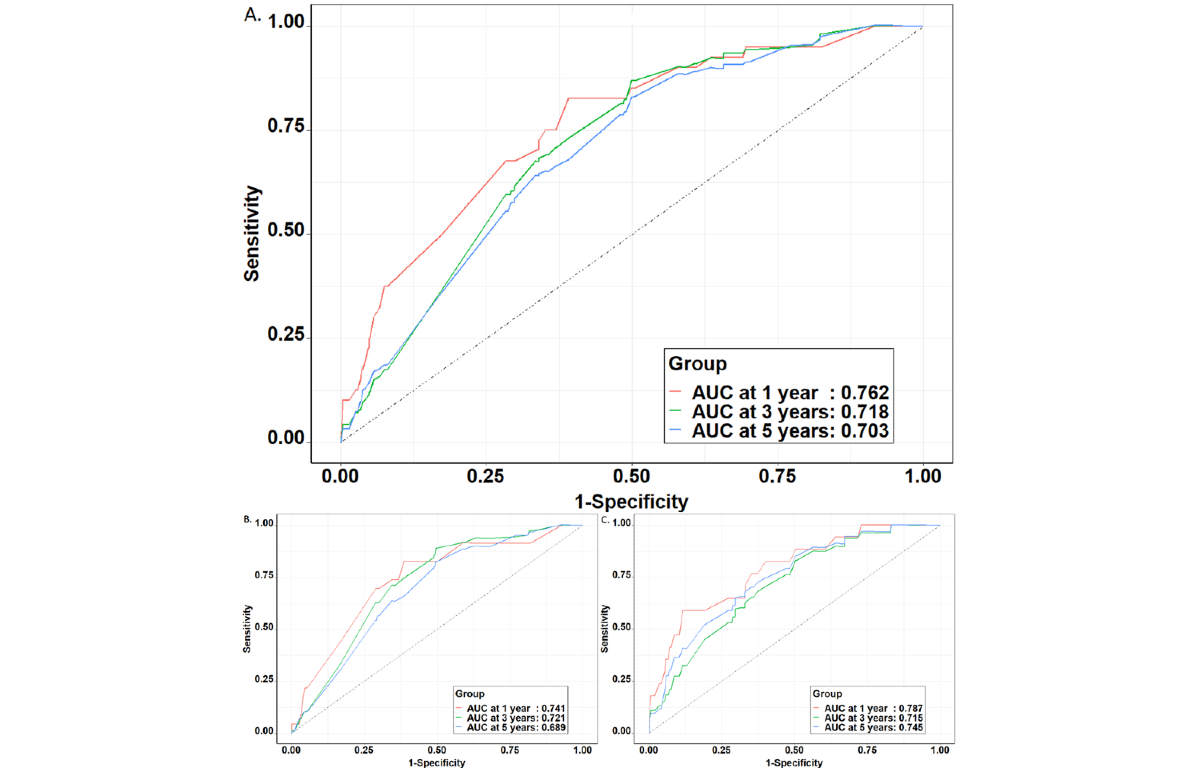
The receiver operating characteristic curves of prediction models in the training set. (A) Whole population; (B) Nonpassive smokers; (C) Passive smokers. AUC: area under the curve.

**Figure 4:**
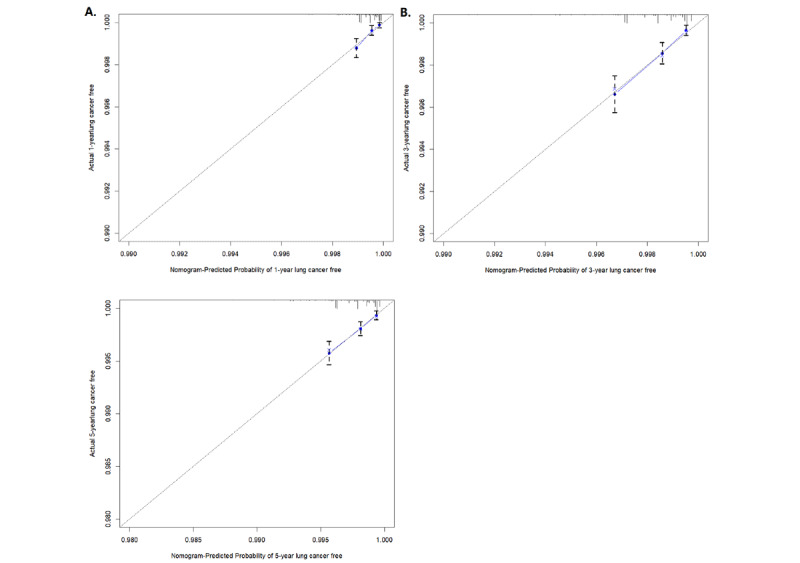
Calibration curves of the nomogram for (A) 1-year, (B) 3-year and (C) 5-year lung cancer–free rates in the training set.

By using this model, the AUC for 1-year, 3-year, and 5-year lung cancer risk in the training set was 0.762, 0.718, and 0.703, respectively. The model yielded a greater AUC for passive smokers (1-year: 0.787, 3-year: 0.715, and 5-year: 0.745) than for nonpassive smokers (1-year: 0.741, 3-year: 0.721, and 5-year: 0.689; Figure 3). Calibration was acceptable, with very similar observed and predicted hazards (Figure 4).

### Validation of the Lung Cancer Risk Model

The model demonstrated a moderate predictive discrimination in the validation set, with AUCs of 0.646, 0.658, and 0.650 for 1-year, 3-year, and 5-year lung cancer risks, respectively (Figure S1 in Multimedia Appendix 1), and satisfactory calibration of relative risk (Figure S2 in Multimedia Appendix 1).

## Discussion

We constructed and validated a simple risk predictive model internally for lung cancer in nonsmoking women relying on 5 commonly accessible factors such as demographics (age), comorbidities (chronic respiratory disease), first-degree family history of lung cancer, and fertility (menopause and history of benign breast disease). Our results showed that the model has moderate discriminatory accuracy and goodness of fit for both nonpassive smokers and passive smokers.

Multiple lung cancer risk variables were discovered for nonsmoking women, such as passive smoking [51,52], prior lung diseases (tuberculosis, chronic bronchitis, emphysema, and prior lung disorders [chronic obstructive pulmonary disease]) [53], indoor radon [54], cooking oil fume [55], and a family history of lung cancer [56]. The established risk variables for lung cancer, such as age, a family history of lung cancer, and a history of chronic respiratory disease, are similar to the findings. Age is the most important risk variable for lung cancer in nonsmoking women according to our survey, which found that the risk was more than 2.4 times higher in the age group of 65-69 years than 40-44 years.

Menopause was associated with an increased risk of developing lung cancer, with an overall odds ratio of 1.33 (95% CI 0.90-1.96), according to a pooled analysis of nested case-control data [57], which is consistent with our findings. Interestingly, we found that women with a history of benign breast disease were less likely to develop lung cancer, possibly because these women may be more careful about their lifestyle and diet after developing breast disease than those who did not. This finding will need to be validated in future studies.

Besides the accurate indicators, risk predicting models should achieve performance standards for discrimination (the differentiation capacity to distinguish lung cancer cases from control ones) and calibration (defined as the consistency between observed and predicted risk for lung cancer). Since 2010, the substantial growth in the numbers of investigations on lung cancer risk predicting models shows the necessity of using predictive models to drive population triage. Initially, models, such as the Bach model [12], Spitz model [13], Liverpool Lung Project model [14], and PLCO_M2012_ model [58], emphasized the importance of applying the classic epidemiological risk variables, including age, smoking history, personal history of disease, and family history of cancer. To the best of our knowledge, this study is one of the few studies to model the prediction of lung cancer risk among nonsmoking Chinese women. Due to the fact that each model was created in a distinct population with different baseline risks and lengths of follow-up, it is challenging to compare the discriminating performance of risk predictive models. The discriminating ability of every model was quite equal, with C-statistics ranging between 0.72 and 0.86. Compared to prior research, our models showed comparable predictive performance.

In understanding our findings, certain strengths and limitations should be carefully considered. Our research is conducted on a large population-based cancer-screening program in mainland China, which is a strong point. In addition, the variables included in this model could be easily collected and updated without any imaging, sophisticated testing, or calculation. Furthermore, the model will be used as a convenient method to triage high-risk people among nonsmoking women, and it will be involving in public health initiatives, such as recommendations regarding the control of lung cancer in nonsmokers. Nonetheless, the statistics based on self-report may be susceptible to social acceptability bias as well as recall bias. Since data collection and quality control were carried out to a high standard, the vast volume of information can be relied upon. Second, the performance of our risk-predicting model was not validated against an external data set before it was used. The findings of the internal calibration, on the other hand, suggest that this model will function satisfactorily when applied to a variety of populations.

In conclusion, a large-scale lung cancer–screening project in China served as the foundation for the creation and internal calibration of a straightforward risk predictive model for lung cancer in nonsmoking women. The model has moderate discrimination and could be used as a tool for triaging high-risk people to prevent lung cancer in nonsmoking women. To validate the concept in external populations, additional prospective studies are needed.

## Appendix

Multimedia Appendix 1 Supplementary table and figures.

## Abbreviations

AUC: area under the curve
CanSPUC: Cancer Screening Program in Urban China
HR: hazard ratio
PLCO: Prostate, Lung, Colorectal, and Ovarian Cancer Screening Trial

## Data Availability

The data sets for this manuscript are not publicly available because all our data are under regulation of both the National Cancer Center of China and The Affiliated Cancer Hospital of Zhengzhou University and Henan Cancer Hospital. Requests to access the data sets should be directed to SZ.
