# Supplementary material for: Lung Cancer Risk Prediction Nomogram in Nonsmoking Chinese Women: Retrospective Cross-sectional Cohort Study
Source: JMIR Public Health Surveill. 2023 Jan 6;9:e41640. doi: 10.2196/41640 (PMC9862335; doi:10.2196/41640)
Supplement: Multimedia Appendix 1 [file publichealth_v9i1e41640_app1.docx]

**Multimedia Appendix 1-Supplemental Materials**

**Lung Cancer Risk Prediction Nomogram in Nonsmoking Chinese**

**Women: Retrospective Cross-sectional Cohort Study**

Lan-Wei Guo^1^, Qing-Cheng Meng^2^, Li-Yang Zheng^1^, Qiong Chen^1^, Yin Liu^1^, Hui-Fang Xu^1^, Rui-Hua Kang^1^, Lu-Yao Zhang^1^, Shu-Zheng Liu^1^, Xi-Bin Sun^1^, Shao-Kai Zhang^1^

^1^ Department of Cancer Epidemiology and Prevention, Henan Engineering Research Center of Cancer Prevention and Control, Henan International Joint Laboratory of Cancer Prevention, The Affiliated Cancer Hospital of Zhengzhou University & Henan Cancer Hospital, Zhengzhou 450008, China

^2^ Department of Radiology, The Affiliated Cancer Hospital of Zhengzhou University & Henan Cancer Hospital, Zhengzhou 450008, China

*Corresponding authors. Dr. Shaokai Zhang, Email: shaokaizhang@126.com

**This file includes:**

Table S1

Figure S1 to Figure S2

**Table S1 Baseline characteristics of the study population.** Compared with participants without lung cancer, lung cancer cases were more likely to have an elder age, have a history of chronic respiratory disease, have a first-degree family history of lung cancer and menopause (all *P* vales <0.05).

| **Variables** | **Total no. (%)** | **Non-lung cancer, n (%)** | **Lung cancer, n (%)** | **χ^2^** | ***P-*value** |
| --- | --- | --- | --- | --- | --- |
| All participants | 151834 | 151630 (99.87) | 204 (0.13) |  |  |
| Person-years, median(IQR) | 2.95 (1.72-4.83) | 2.95 (1.72-4.83) | 1.52 (0.84-2.62) |  |  |
| ***Demographic characteristics*** |  |  |  |  |  |
| Age, mean±SD, years | 55.34±8.66 | 55.33±8.66 | 60.20±7.60 |  |  |
| Age (years) |  |  |  | 65.93 | **<.001** |
| 40-44 | 18614 (12.26) | 18607 (99.96) | 7 (0.04) |  |  |
| 45-49 | 27362 (18.02) | 27345 (99.94) | 17 (0.06) |  |  |
| 50-54 | 28574 (18.82) | 28549 (99.91) | 25 (0.09) |  |  |
| 55-59 | 23572 (15.52) | 23540 (99.86) | 32 (0.14) |  |  |
| 60-64 | 25874 (17.04) | 25818 (99.78) | 56 (0.22) |  |  |
| 65-69 | 20381 (13.42) | 20329 (99.74) | 52 (0.26) |  |  |
| 70-74 | 7457 (4.91) | 7442 (99.80) | 15 (0.20) |  |  |
| Race |  |  |  | 0.25 | .62 |
| Han nationality | 148870 (98.05) | 148669 (99.86) | 201 (0.14) |  |  |
| Others | 2964 (1.95) | 2961 (99.90) | 3 (0.10) |  |  |
| Education ^a^ |  |  |  | 1.48 | .48 |
| Low | 32269 (21.25) | 32222 (99.85) | 47 (0.15) |  |  |
| Medium | 99829 (65.75) | 99693 (99.86) | 136 (0.14) |  |  |
| High | 19736 (13.00) | 19715 (99.89) | 21 (0.11) |  |  |
| Marriage |  |  |  | 2.56 | .11 |
| Unmarried/divorce/widowed | 6386 (4.21) | 6382 (99.94) | 4 (0.06) |  |  |
| Married | 145448 (95.79) | 145248 (99.86) | 200 (0.14) |  |  |
| BMI (kg/m^2^) |  |  |  | 0.96 | .81 |
| <18.5 | 2280 (1.50) | 2277 (99.87) | 3 (0.13) |  |  |
| 18.5-24.0 | 70979 (46.75) | 70883 (99.86) | 96 (0.14) |  |  |
| 24.0-28.0 | 61252 (40.34) | 61166 (99.86) | 86 (0.14) |  |  |
| ≥28.0 | 17323 (11.41) | 17304 (99.89) | 19 (0.11) |  |  |
| ***Dietary habit*** |  |  |  |  |  |
| Vegetables intake |  |  |  | 0.16 | .69 |
| ≥2.5kg/week | 78798 (51.90) | 78695 (99.87) | 103 (0.13) |  |  |
| <2.5kg/week | 73036 (48.10) | 72935 (99.86) | 101 (0.14) |  |  |
| Fruit intake |  |  |  | 0.05 | .83 |
| ≥1.25kg/week | 87478 (57.61) | 87362 (99.87) | 116 (0.13) |  |  |
| <1.25kg/week | 64356 (42.39) | 64268 (99.86) | 88 (0.14) |  |  |
| Roughage intake |  |  |  | 2.32 | .13 |
| ≥0.5kg/week | 103550 (68.20) | 103421 (99.88) | 129 (0.12) |  |  |
| <0.5kg/week | 48284 (31.80) | 48209 (99.84) | 75 (0.16) |  |  |
| ***Living environment, behavior and habits*** |  |  |  |  |  |
| Cooking oil fume exposure |  |  |  | 0.49 | .49 |
| None or a little | 131445 (86.57) | 131265 (99.86) | 180 (0.14) |  |  |
| A lot | 20389 (13.43) | 20365 (99.88) | 24 (0.12) |  |  |
| Passive smoking |  |  |  | 1.49 | .22 |
| No | 98006 (64.55) | 97866 (99.86) | 140 (0.14) |  |  |
| Yes | 53828 (35.45) | 53764 (99.88) | 64 (0.12) |  |  |
| Alcohol Drinking |  |  |  | 1.88 | .39 |
| Never | 143282 (94.37) | 143085 (99.86) | 197 (0.14) |  |  |
| Current | 7126 (4.69) | 7120 (99.92) | 6 (0.08) |  |  |
| Former | 1426 (0.94) | 1425 (99.93) | 1 (0.07) |  |  |
| Physical activity |  |  |  | 9.71 | **.002** |
| Moderate or no | 80530 (53.04) | 80444 (99.89) | 86 (0.11) |  |  |
| Heavy | 71304 (46.96) | 71186 (99.83) | 118 (0.17) |  |  |
| ***Psychology and emotions*** |  |  |  |  |  |
| History of a severe trauma |  |  |  | 0.46 | .50 |
| No | 130517 (85.96) | 130345 (99.87) | 172 (0.13) |  |  |
| Yes | 21317 (14.04) | 21285 (99.85) | 32 (0.15) |  |  |
| Mental depression for over 6 months |  |  |  | 0.04 | .85 |
| No | 128783 (84.82) | 128609 (99.86) | 174 (0.14) |  |  |
| Yes | 23051 (15.18) | 23021 (99.87) | 30 (0.13) |  |  |
| ***Comorbidities*** |  |  |  |  |  |
| History of chronic respiratory disease |  |  |  | 7.58 | **.006** |
| No | 128200 (84.43) | 128042 (99.88) | 158 (0.12) |  |  |
| Yes | 23634 (15.57) | 23588 (99.81) | 46 (0.19) |  |  |
| History of tuberculosis |  |  |  | 4.29 | **.04** |
| No | 149861 (98.70) | 149663 (99.87) | 198 (0.13) |  |  |
| Yes | 1973 (1.30) | 1967 (99.70) | 6 (0.30) |  |  |
| History of chronic bronchitis |  |  |  | 3.22 | .07 |
| No | 133482 (87.91) | 133311 (99.87) | 171 (0.13) |  |  |
| Yes | 18352 (12.09) | 18319 (99.82) | 33 (0.18) |  |  |
| History of emphysema |  |  |  | 2.55 | .11 |
| No | 150463 (99.10) | 150263 (99.87) | 200 (0.13) |  |  |
| Yes | 1371 (0.90) | 1367 (99.71) | 4 (0.29) |  |  |
| History of asthma bronchiectasis |  |  |  | 0.36 | .55 |
| No | 146998 (96.81) | 146802 (99.87) | 196 (0.13) |  |  |
| Yes | 4836 (3.19) | 4828 (99.83) | 8 (0.17) |  |  |
| History of hypertension |  |  |  | 3.26 | .07 |
| No | 122222 (80.50) | 122068 (99.87) | 154 (0.13) |  |  |
| Yes | 29612 (19.50) | 29562 (99.83) | 50 (0.17) |  |  |
| History of hyperlipidemia |  |  |  | 1.33 | .25 |
| No | 126622 (83.40) | 126458 (99.87) | 164 (0.13) |  |  |
| Yes | 25212 (16.60) | 25172 (99.84) | 40 (0.16) |  |  |
| History of diabetes |  |  |  | 8.04 | **.005** |
| No | 141542 (93.22) | 141362 (99.87) | 180 (0.13) |  |  |
| Yes | 10292 (6.78) | 10268 (99.77) | 24 (0.23) |  |  |
| ***First-degree family history of lung cancer*** |  |  |  | 6.58 | **.01** |
| No | 139835 (92.10) | 139657 (99.87) | 178 (0.13) |  |  |
| Yes | 11999 (7.90) | 11973 (99.78) | 26 (0.22) |  |  |
| ***Physiology and fertility*** |  |  |  |  |  |
| Age of menarche (y) |  |  |  | 2.02 | .16 |
| <12 | 3868 (2.55) | 3866 (99.95) | 2 (0.05) |  |  |
| ≥12 | 147965 (97.45) | 147763 (99.86) | 202 (0.14) |  |  |
| Menopause |  |  |  | 52.69 | **<.001** |
| No | 54039 (35.59) | 54016 (99.96) | 23 (0.04) |  |  |
| Yes | 97795 (64.41) | 97614 (99.81) | 181 (0.19) |  |  |
| Fertility status |  |  |  | 2.79 | .10 |
| No | 2044 (1.35) | 2044 (100.00) | 0 (0.00) |  |  |
| Yes | 149790 (98.65) | 149586 (99.86) | 204 (0.14) |  |  |
| Lactation status |  |  |  | 1.08 | .30 |
| No | 8485 (5.59) | 8477 (99.91) | 8 (0.09) |  |  |
| Yes | 143349 (94.41) | 143153 (99.86) | 196 (0.14) |  |  |
| History of benign breast disease |  |  |  | 4.61 | **.03** |
| No | 108002 (71.13) | 107843 (99.85) | 159 (0.15) |  |  |
| Yes | 43832 (28.87) | 43787 (99.90) | 45 (0.10) |  |  |
| History of reproductive system surgery |  |  |  | 0.10 | .76 |
| No | 121161 (79.80) | 121000 (99.87) | 161 (0.13) |  |  |
| Yes | 30673 (20.20) | 30630 (99.86) | 43 (0.14) |  |  |

a. Low, primary school or below; Medium, junior or senior high school; High, undergraduate or over.

Abbreviations: IQR, Interquartile range; BMI, body mass index.

**Figure S1. The receiver operating characteristic curves of prediction models in the validation set.** The model showed a good predictive discrimination in the validation set, with the AUC was 0.646, 0.658, and 0.650 for 1-year, 3-year, and 5-year lung cancer risk.

**
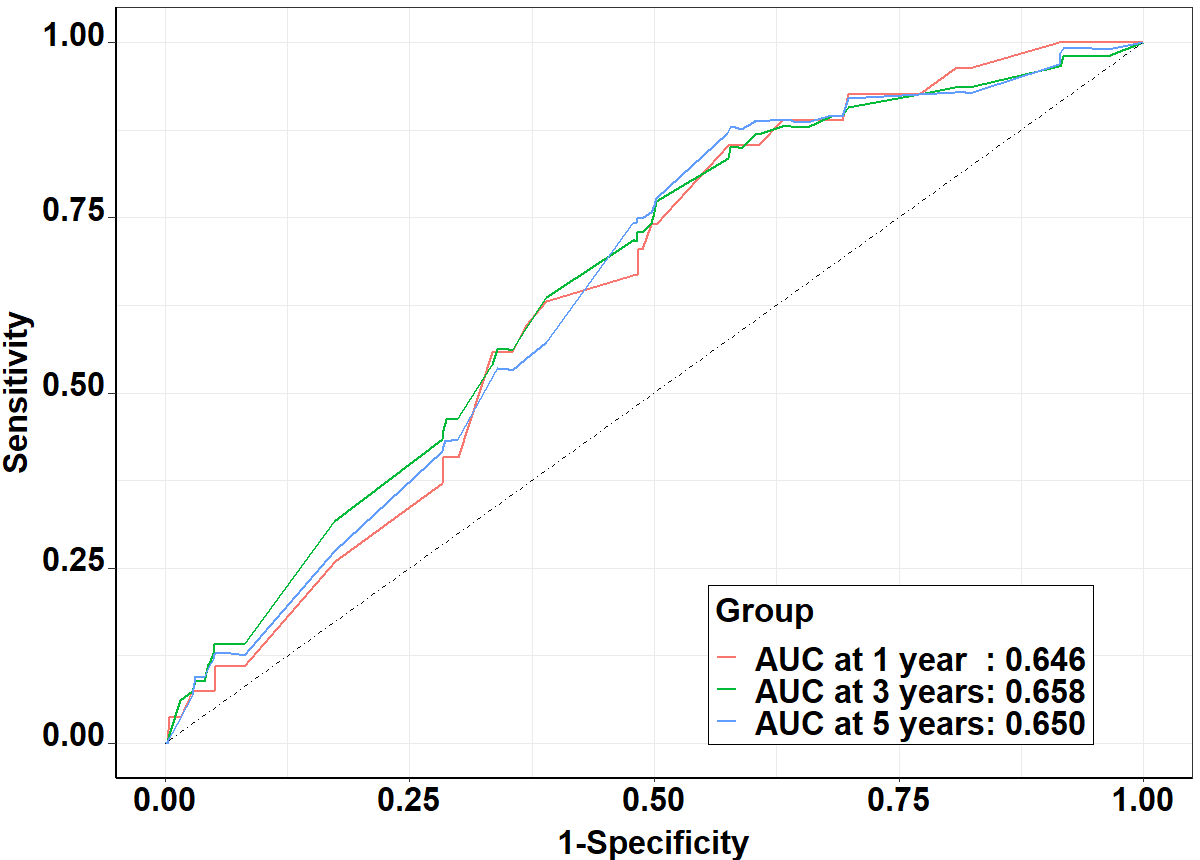
**

**Figure S2. Calibration curves of the nomogram for (A) 1-year, (B) 3-year and (C) 5-year lung cancer free in the validation set.** The model showed the satisfactory calibration of relative risk.

**
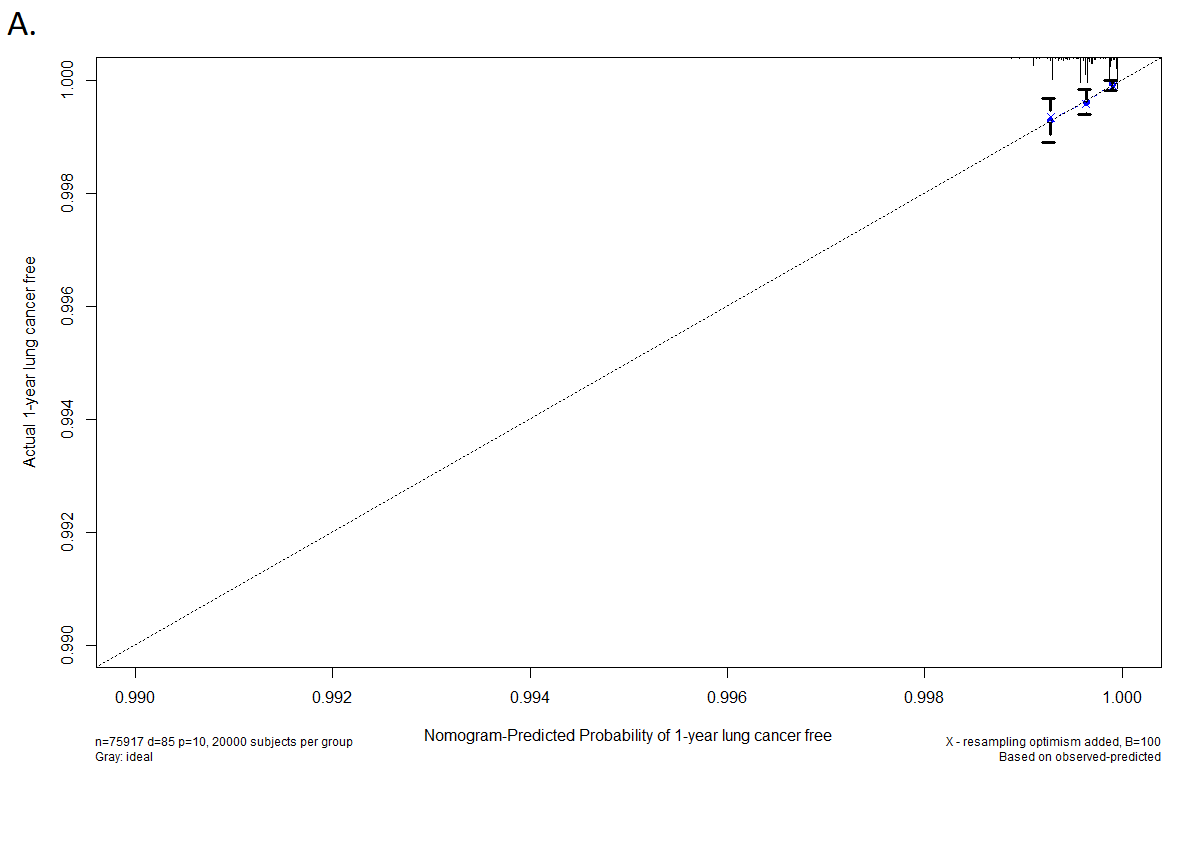
**

**
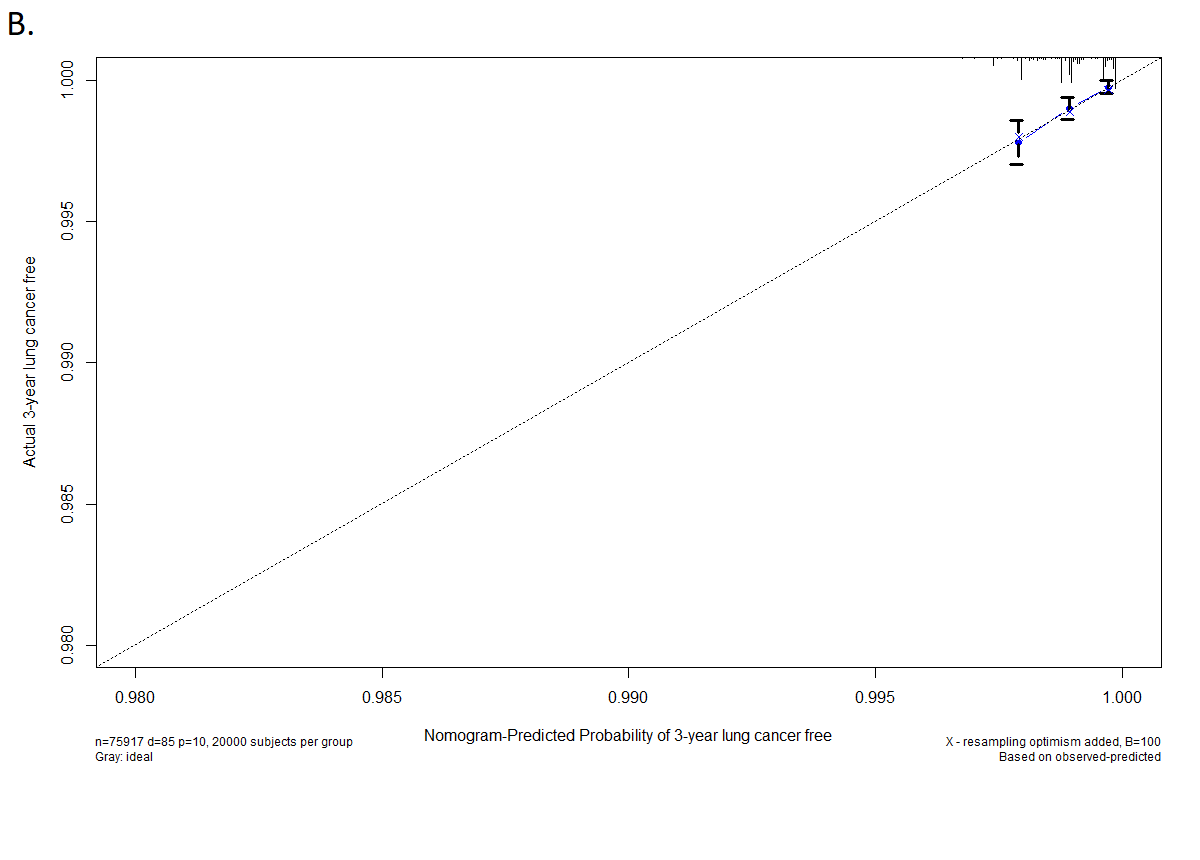
**

**
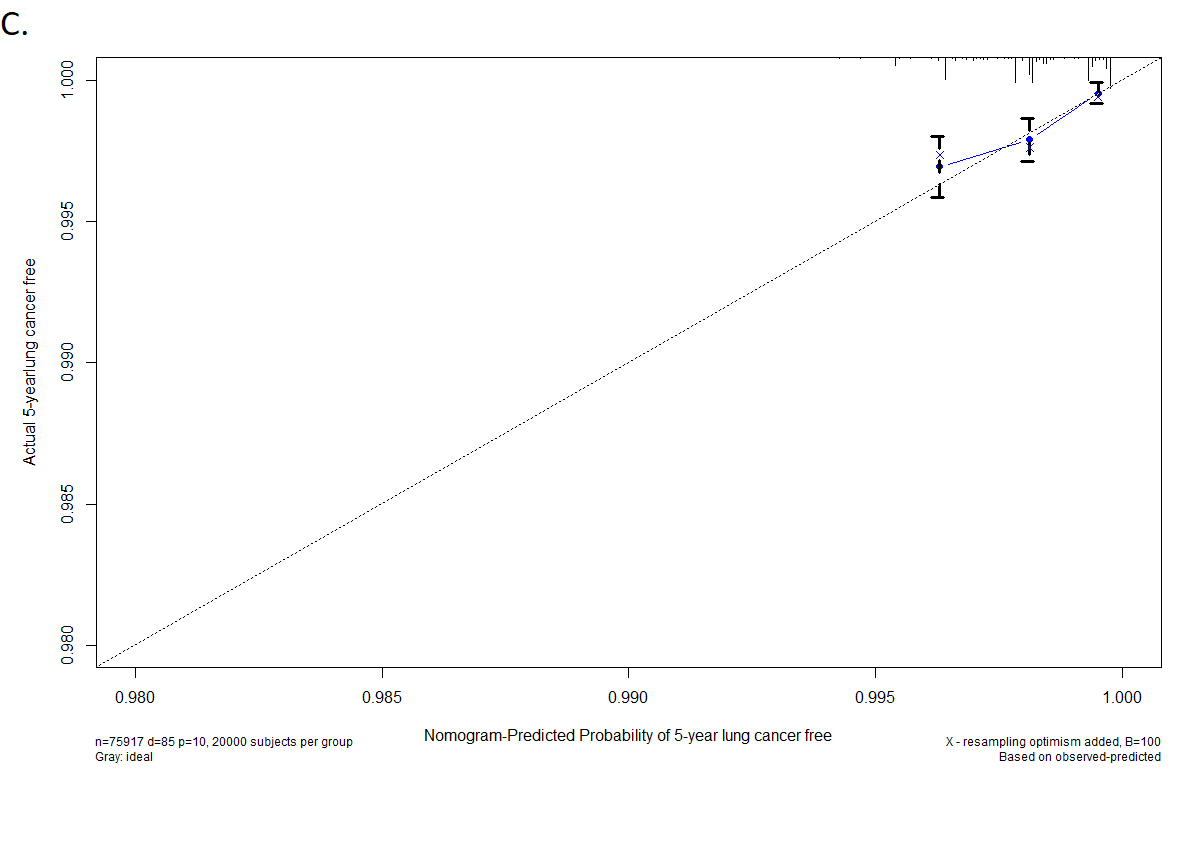
**
